# Chimeric antigen receptor-engineered NK cells: new weapons of cancer immunotherapy with great potential

**DOI:** 10.1186/s40164-022-00341-7

**Published:** 2022-11-02

**Authors:** Xiao Wang, Xuejiao Yang, Xiang Yuan, Wenbo Wang, Yueying Wang

**Affiliations:** 1 Shanghai Institute of Hematology, State Key Laboratory of Medical Genomics, National Research Center for Translational Medicine at Shanghai, Ruijin Hospital, Shanghai Jiao Tong University School of Medicine, Shanghai, 200025 China; 2 Department of Thoracic Oncology, Cancer Center and State Key Laboratory of Biotherapy, West China Hospital, Sichuan University, Chengdu, 610041 China; 3 Department of Oncology, Shanghai Tenth People’s Hospital, Tongji University School of Medicine, Shanghai, 200072 China

**Keywords:** T cells, Natural killer cells, Chimeric antigen receptor, Cancer, Cellular immunotherapy

## Abstract

Chimeric antigen receptor (CAR)-engineered T (CAR-T) cells have obtained prominent achievement in the clinical immunotherapy of hematological malignant tumors, leading to a rapid development of cellular immunotherapy in cancer treatment. Scientists are also aware of the prospective advantages of CAR engineering in cellular immunotherapy. Due to various limitations such as the serious side effects of CAR-T therapy, researchers began to investigate other immune cells for CAR modification. Natural killer (NK) cells are critical innate immune cells with the characteristic of non-specifically recognizing target cells and with the potential to become “off-the-shelf” products. In recent years, many preclinical studies on CAR-engineered NK (CAR-NK) cells have shown their remarkable efficacy in cancer therapy and their superiority over autologous CAR-T cells. In this review, we summarize the generation, mechanisms of anti-tumor activity and unique advantages of CAR-NK cells, and then analyze some challenges and recent clinical trials about CAR-NK cells therapy. We believe that CAR-NK therapy is a promising prospect for cancer immunotherapy in the future.

## Introduction

Cellular immunotherapy plays an indispensable role in cancer treatment. Chimeric antigen receptor (CAR)–engineered T cells therapy, especially for the treatment of hematological malignant tumors, has become a research hotspot over the past decade. One BCMA-targeted and four CD19-targeted CAR-T products have been approved for marketing by the American Food and Drug Administration (FDA): tisagenlecleucel and axicabtagene ciloleucel for the treatment of relapsed/refractory (R/R) large B-cell lymphoma and pediatric B-cell acute lymphocytic leukemia, brexucabtagene autoleucel for R/R mantle cell lymphoma, lisocabtagene maraleucel for R/R large B-cell lymphoma, and idecabtagene vicleucel for R/R multiple myeloma [1–5]. However, CAR-T therapy still has many unavoidable limitations that hinder further development in clinical treatment (Table 1): (1) The safety of CAR-T cells needs to be solved. Cytokine release syndrome (CRS) and immune effector cell-associated neurotoxicity syndrome (ICANS) are the major inevitable toxicities that still lack corresponding effective management in most cases [6]. (2) CAR-T therapy requires T cells that can only be derived and engineered from the autologous peripheral blood and then injected back into the patient, so it is time-consuming and expensive for patients. (3) T cells are the main effector cells of graft-versus-host disease (GVHD), so CAR-T therapies have the risk of developing GVHD, which requires higher safety of CAR-T products [7]. (4) Other limitations include the off-targeted toxicity and the ineffective treatment of solid tumors due to the immunosuppressive microenvironment [8–11]. Given the shortcomings of CAR-T therapy, researchers began to apply the principles of CAR-engineered therapy to other immune cells [12, 13]. Because of the unique biological characteristics, anti-tumor mechanisms, wide range of sources, and higher safety of natural killer (NK) cells, CAR-engineered NK (CAR-NK) cells which are regarded as a promising alternative platform have attracted considerable attention in recent years [13–17]. In this review, we will discuss CAR-NK cells in detail, including the generation, tumor-killing mechanisms and promising advantages of CAR-NK cells, and then analyze some challenges and recent clinical trials about CAR-NK cells therapy.

**Table 1:** Comparison of the advantages and disadvantages of CAR-T therapy and CAR-NK therapy

	CAR-T therapy	CAR-NK therapy
Advantage	Better expansion and persistence Easier transduction and modification Proven prospects for clinical use (many positive clinical results have been reported; CAR-T clinical products approved by FDA)	Various allogeneic sources (PB, UCB, NK cell lines, iPSC) Good safety, no GVHD, CRS and ICANS CAR-dependent killing mechanism and innate cytotoxicity (including ADCC) “Off-the-shelf” products availability and lower cost
Disadvantage	Single source (patient's autologous T cells) Risk of GVHDs and side effects (CRS, ICANS) Only CAR-dependent killing mechanism Higher cost and low production efficiency (needs to be prepared individually for each patient) Low potential of “off-the-shelf” products	Difficulty in large-scale expansion and short lifespan More difficult to transduce and modify More encouraging clinical results are still needed to prove its application prospects

## Generation of CAR-NK cells

**Fig. 1:**
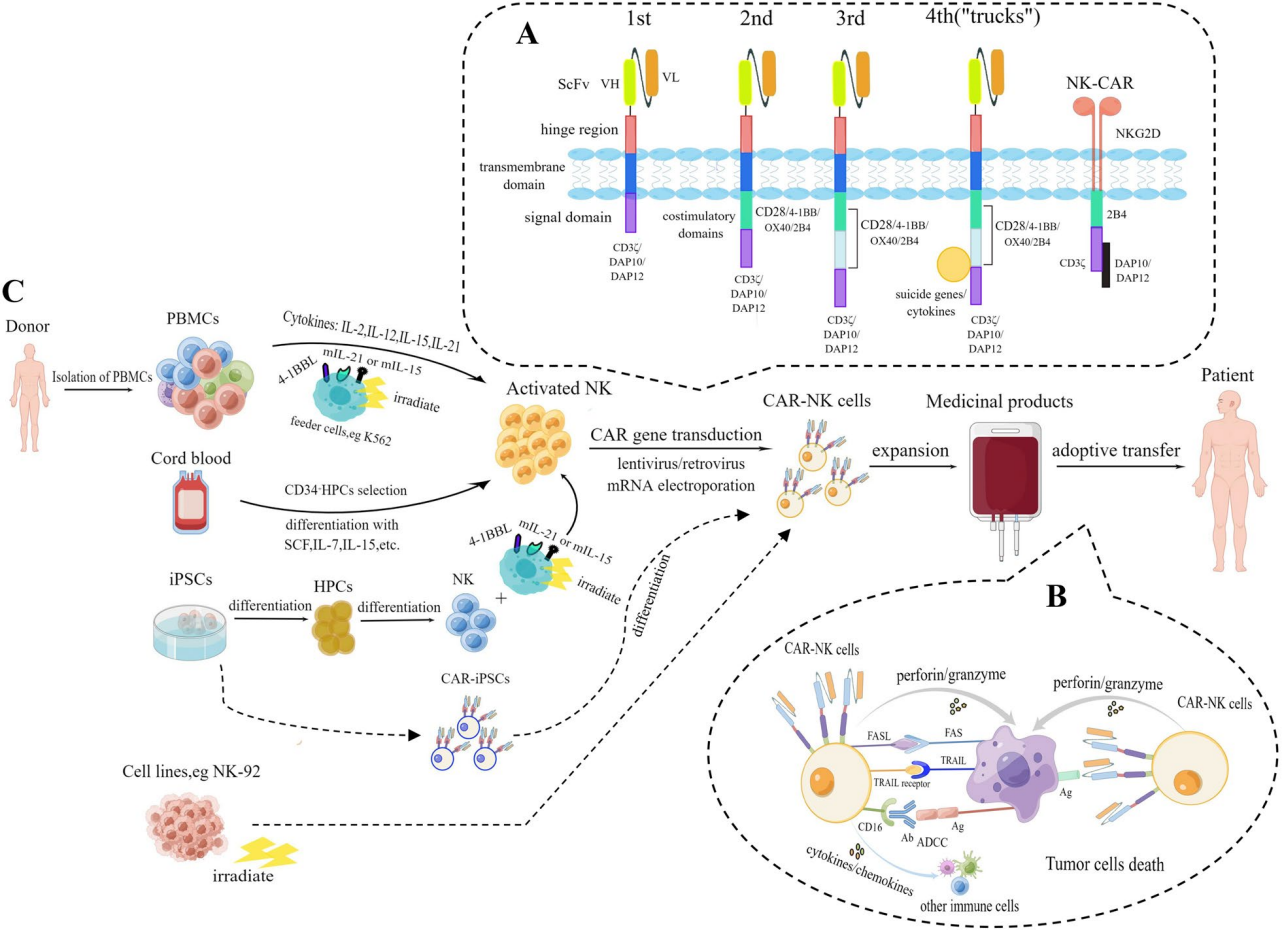
Generation of CAR-NK cells for adoptive transfer immunotherapy in cancer patients. **A**. Evolution of CAR structures from the first generation to the fourth generation according to NK cell intracellular motifs and functions. The new NK-CAR structure contains NKG2D ectodomain with CD3ζ, DAP10 or DAP12 cytoplasmic signal domain. **B**. The mechanisms of tumor destruction by CAR-NK cells through both CAR-dependent and CAR-independent manners. **C**. Different cell sources and corresponding procedures for manufacturing CAR-NK cell products

The success of CAR-T therapy in clinical trials has led to the development of CAR-NK cells. Genetic engineering of NK cells with a CAR structure is also similar with the generation of CAR-T cells. The procedure for adoptive CAR-NK therapy in cancer patients is described in the Fig. 1.

### CAR structures

At present, most preclinical studies on CAR-NK cells still follow the CAR structures adopted by CAR-T therapies. The CAR construct is similar to that of the T cell receptor. It consists of several parts including an extracellular antigen binding region, an extracellular hinge domain and a transmembrane region, and an intracellular signal transduction region that transmits activation signals and costimulatory signals to T cells. The extracellular antigen binding region is a single-chain variable fragment (scFv), which is constructed by connecting the heavy chain variable region and the light chain variable region of the monoclonal antibody via a short linker peptide [18–20]. The CAR structure has undergone at least five generations of evolution. The first-generation structure that only provides the first activation signal for T cells cannot effectively activate T cells and provide a continuous anti-tumor effect in vivo [21, 22]. In the second- or third-generation structures, one or two costimulatory signal molecules are added to provide the second activation signal. CD28 and 4-1BB (CD137) are two commonly used classical costimulatory domains [23], and others such as CD27, OX40 (CD134), ICOS (CD278), CD40, toll-like receptors, and complement 3a receptor (C3aR) have also been studied [24–28]. In the fourth generation, some structures such as suicide genes and cytokines are integrated to regulate T cells precisely, which is worthwhile exploring to reshape the tumor microenvironment (TME) [29–31]. In the new generation of CAR-T cells, endogenous T cell receptors and leukocyte antigen class I (HLA) are knocked out and fusion proteins are integrated for reducing the toxic side effects and re-establishing the immune system after infusion [20].

Previous studies of CAR-NK cells used the first four generations of CAR constructs designed for T cells, such as 4-1BB, CD28 costimulatory domains, and CD3ζ signaling domains. However, recent studies have shown that the domains specific to NK cells can produce stronger activation of NK cells and anti-tumor response [32]. Ye Li and his team demonstrated that CAR constructs specifically designed to enhance NK cell activity have a potent ability to kill tumors both in vitro and in vivo. They finally concluded that CAR-NK cells with scFv (anti-mesothelin)-NKG2D-2B4-CD3ζ (NK-CAR4 structure) have stronger cytotoxicity, expansibility and persistence than scFv-CD28-CD137-CD3ζ (T-CAR structure) [33]. CD3ζ and DNAX-activation proteins (DAP10 and DAP12) are signaling adaptor molecules that contain immunoreceptor tyrosine-based activation motifs (ITAMs). They can initialize the activation of NK cells through phosphorylation mediated by protein tyrosine kinases of the Src family and then up-regulate the phosphorylation of signaling pathways such as the Syk-vav1-Erk and NF-kB pathways, resulting in cytotoxicity (release of cytotoxic granules, including perforin and granzymes) and cytokines production (e.g., TNF-α and IFN-γ) [34–36]. As an important activation receptor, NKG2D is a C-type lectin-like family molecule expressed on the surface of almost all NK cells [37]. DAP10 is a downstream signal molecule that transmits signals for the activating receptor NKG2D. The two can combine specifically via the induced fit theory and further induce phosphorylation [38, 39]. Similarly, DAP12 transmits activation signals for activating receptors such as NKG2C and NKp44. The DAP12 domain that contains only one ITAM can provide sufficient and better activation signaling to NK cells compared with a CAR containing the CD3ζ chain with three ITAMs [40]. Another activating receptor, 2B4, belongs to the signaling lymphocytic activation molecule family and contains an immunoreceptor tyrosine-based switch motif that mediates signal transduction associated with the activation of NK cells. And 2B4 was demonstrated to enhance cytokine secretion after linking with NKG2D [41]. The evolution of the CAR structures and the specific NK-CAR were displayed in the Fig. 1A.

### CAR vector transfer in NK cells

**Fig. 2:**
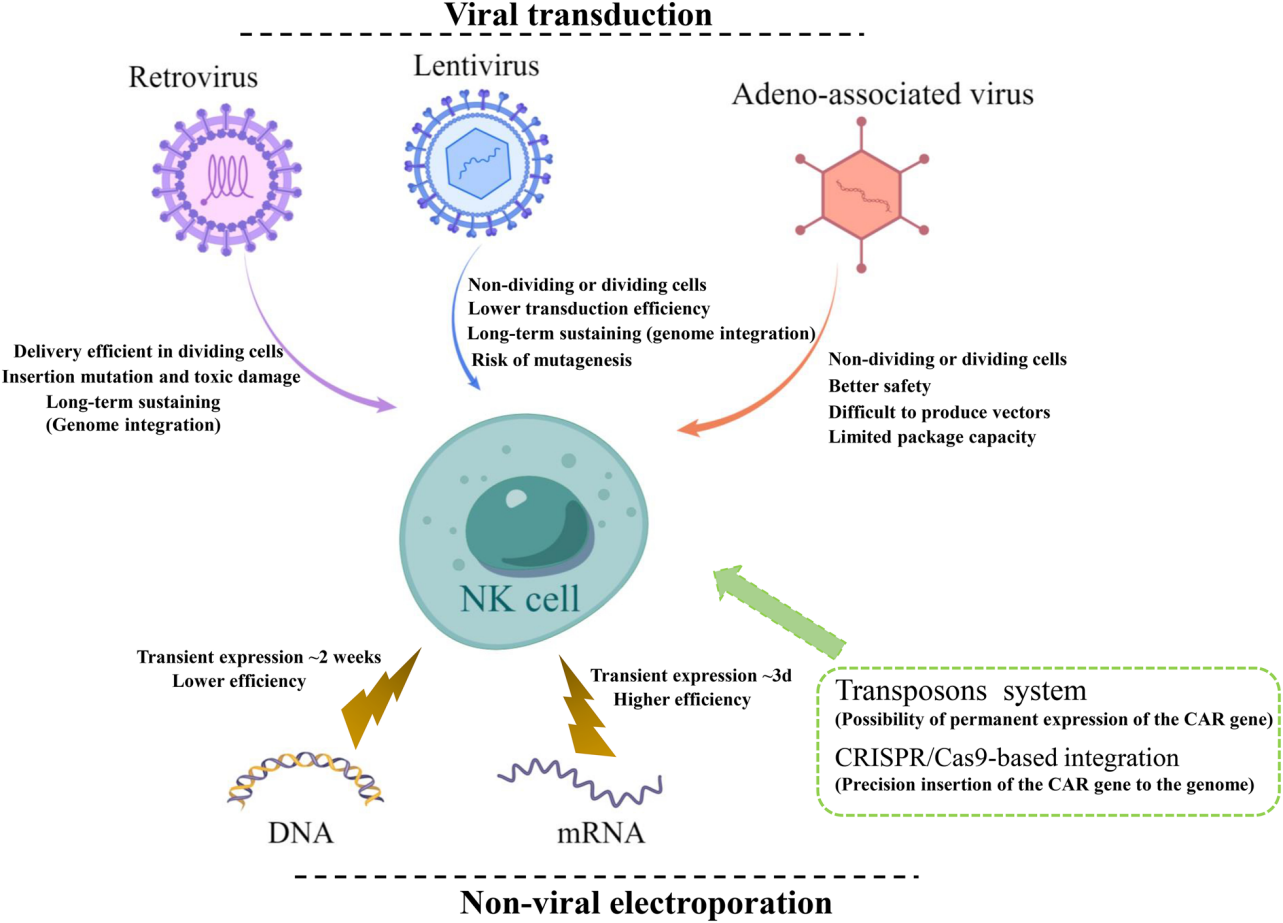
Various approaches of delivering CAR into NK cells. The methods for genetic engineering of NK cells including viral transduction (upper) and non-viral electroporation (lower), as well as their strengths and drawbacks are illustrated

NK cells are more difficult to be successfully engineered with CAR structures compared with T cells, as a result of different sensitivity to the interference of foreign introduced genes [42]. Thus, it leads to lower transduction efficiency than CAR engineered to T cells due to the resistance of inserting foreign genes mediated by their natural defense receptors [43]. Approaches for CAR gene transferred into NK cells can be divided into viral and non-viral ways (Fig. 2). And currently, there is no available gene transfer method that is universally applicable because every method more or less has flaws.

Viral transductions including the retroviral and lentiviral-based vectors enable foreign genes to insert into the targeted cell genome and stably express for a long time. Viral vectors can accommodate complicated and long sequences up to 10kbp, and transfer them into the host integrally [44]. It is also the main method used in a large number of CAR-NK preclinical studies. However, there are likewise some drawbacks of viral transduction. Retroviral vectors are likely to cause insertion mutation and toxic damage to primary NK cells, and can only introduce foreign genes when the host cells are in the stage of proliferation with their nuclear membrane dissolving [45]. Before transduction, NK cells need to be stimulated into an expanded state with some cytokines (e.g., IL-2, IL-21, IL-15, IL-12, IL-18) [46, 47]. In contrast to retroviral vectors, lentiviral vectors can integrate their genetic materials into resting cells but with lower efficiency. Multiple rounds of transfection are often needed to achieve the transduction efficiency required [48, 49]. In order to improve the efficiency, it is often necessary to add auxiliary transfection reagents, such as polybrene, RetroNectin, or Vectofusin-1[50–52]. Müller and his coworkers compared two different vectors, lentiviral and alpharetroviral, both combined with two different transduction enhancers (RetroNectin and Vectofusin-1). They concluded that using RD114-TR pseudotyped retroviral particles in combination with Vectofusin-1 is a promising strategy to genetically modify NK cells to achieve highly cytotoxic CD19-CAR-NK cells with high yield [53]. In addition, it can also optimize virus packaging by selecting the best pseudotyping envelope protein, such as using baboon envelop glycoprotein (BaEV-gp) instead of the commonly used vesicular stomatitis virus glycoprotein G (VSV-G). A research group reported that using BaEV pseudotyped lentiviral vectors can obtain a transduction rate of about 83.4% in activated NK-cells, which outperformed VSV-G-, RD114- and measles virus-pseudotyped lentivirus [54]. Another group found that spinfection transduction (centrifugation at a low speed) showed better efficiency (19–73%) with CD19-CAR lentiviral transduced to NK cells from cord blood compared with static transduction (12–30%) [55]. Thus, spinfection may be another method to further optimize viral transduction. Recently, recombinant adeno-associated virus (AAV) has been broadly adopted and been regarded as an alternative gene delivery tool, owing to their high efficiency of transduction and a better safety profile. The combination of CRISPR/Cas9 and AAV resulted in highly efficient and stable CD33-CAR expression applicable to cancer immunotherapy. But AAV also has limitations of constrained packaging capacity and vector production difficulty [56].

Another non-viral strategy of CAR-NK engineering is transfection with electroporation. Electroporation can transport mRNA or DNA into the cell by utilizing short electric pulses to make tiny holes of the cell membrane. But it may damage cells due to membrane leakage [57, 58]. DNA electroporation needs higher transfection conditions and leads to the lower efficiency and viability of cells than mRNA electroporation [59, 60], so it has not been used in clinical trials of CAR-NK cells. Compared with viral transduction, although the efficiency of mRNA electroporation is higher, the expression of the CAR gene cannot be maintained for a long time owing to transient transfection without integrating into the cell genome [61, 62]. And Boissel, L concluded that lentiviral vectors should be used for CAR transduction if primary NK cells are considered. Transfection with mRNA only is suitable for meeting clinical demand in NK-92 cells [55]. Lin Xiao et al. reported that utilizing optimized mRNA electroporation for NK cells expressing NKG2D-DAP12 CAR structures provided almost 99% transfection efficiency, which was maintained for about 5 days [63]. Some researchers combine electroporation with transposons or CRISPR/Cas9-based integration [64–66]. The sleeping beauty transposon system for integrating CAR vectors has been tried out in CAR-NK cells [33]. Recently, the new technology of CRISPR/Cas9 can accurately select the site of integration of the CAR gene and perform gene knockout in NK cells, which provides new possibilities for improving CAR-NK cell products [67]. Gurney et al. applied CRISPR/Cas9 genome editing to knock down the CD38 gene during NK expansion with a mean efficiency of 84% and then expressed an affinity optimized CD38-CAR. They found that the cytotoxic potential of CD38 KO-CD38 CAR-NK cells was augmented and proposed a viable immunotherapeutic approach for the treatment of acute myeloid leukemia (AML) [68].

## Mechanisms of cancer killing

### CAR-independent killing mechanism

The cytotoxicity of NK cells is controlled by a series of active and inhibitory receptors expressed on themselves. When encountering tumor cells or other stress conditions, activating receptors on NK cells, such as NKG2D, NKp30, NKp44 and NKp46, can be stimulated to engage with ligands, thus triggering NK activation and mediating their killing activity [69, 70]. On the contrary, some inhibitory receptors such as killer immunoglobulin-like receptors (KIRs) can inhibit the activity of NK cells when they bind to corresponding ligands. For example, our healthy cells express human leukocyte antigen (HLA) ligands that bind to KIRs so that NK cells do not attack them under normal circumstances [71]. In addition, NK cells exert their non-targeted cytotoxicity in a non-major histocompatibility complex restricted manner. First, NK cells kill target cells through similar cytotoxic mechanisms with CD8^+^ cytotoxic T cells, including releasing perforin/granzyme, which leads to target cell lysis; upregulating Fas ligand or TRAIL receptor on their surface to induce tumor cell apoptosis; and releasing cytokines and chemokines to recruit and activate other immune effector cells such as macrophages and dendritic cells [72–74]. Moreover, CD16 (Fc receptor) expressed on NK cells recognizes the Fc segment of immunoglobulin G that binds to the target cells, thereby mediating the cytotoxicity of NK cells against tumors. It is the antibody-dependent cell-mediated cytotoxicity (ADCC) [75].

### CAR-dependent killing mechanism

Although the cytotoxicity of NK cells is non-targeted, NK cells can reach the tumor site where specific antigens are expressed and kill tumors in targeted ways after genetically engineered with the CAR structure. Figure 1B displayed the two kinds of killing mechanisms of CAR-NK cells. Different specific targets of CAR can be selected according to the various tumor types. Table 2 and Table 3 summarize some preclinical researches of the anti-target CAR-NK cell therapy for hematological malignancies and solid tumors, respectively. For the hematological malignant tumors such as lymphomas and leukemias, CD19 is the most commonly used target that is only overexpressed on B-cell malignancies. BCMA, CD138 and CS1 are the targets of multiple myeloma for CAR-NK cells. Others such as CD7, CD123 and CD5 are also the anti-targets for engineered-NK cell therapy (Table 2). For solid tumors, anti-HER2 and anti-EGFR engineered-NK therapy have been reported in several cancers like glioblastoma, breast, and ovarian cancers. Han reported that EGFR-CAR-engineered NK cells displayed enhanced cytolytic capability and production of IFN-γ, which restrained glioblastoma growth and obviously prolonged the survival of tumor-bearing mice [76]. Other antigens including mesothelin, PSCA, GPA7 and EpCAM were explored as CAR-NK cell targets in solid tumors (Table 3).

**Table 2:** Targets of CAR-NK cell-based therapy for hematological malignancies

Target	Tumor	NK cell sources	CAR structures	CAR transfer	Reference	Year
CD19	B-ALL	PB	scFv-4-1BB-CD3ζ	Retrovirus	[77]	2005
CD19	B-cell leukemia	PB	scFv-4-1BB-CD3ζ	mRNA electroporation	[78]	2012
CD19	B-ALL	PB	scFv-CD28-CD3ζ	Lentivirus	[79]	2016
CD19	B-cell precursor Leukemia	PB	scFv-CD28-4-1BB-CD3ζ	Retrovirus	[80]	2016
CD19	B-cell lymphoma	NK-92	scFv-CD28-4-1BB-CD3ζ	Lentivirus	[81]	2017
CD19	B-cell malignancies	UCB	scFv-4-1BB-CD3ζ + iCasp9 + IL15	Retrovirus	[82]	2018
CD19	B-cell leukemia	NK-92	scFv-4-1BB-CD3ζ	Lentivirus	[83]	2020
CD19	B-cell lymphoma and precursor leukemia	PB	scFv-4-1BB-CD3ζ + CXCR4	Lentivirus	[84]	2020
CD19	B-ALL	PB	scFv-CD28-CD3ζ	Retrovirus	[53]	2020
CD19	B-cell lymphoma	UCB	iC9 + scFv-CD28-CD3ζ + IL-15	Retrovirus	[85]	2021
CD19/BCMA	B-cell malignancies	NK-92	scFv-4-1BB-CD3ζ	mRNA electroporation	[86]	2022
CD20	Burkitt lymphoma	PB	scFv-4-1BB-CD3ζ	mRNA electroporation	[87]	2015
CD20	Burkitt lymphoma	PB	scFv-4-1BB-CD3ζ	mRNA electroporation	[88]	2017
CD138	multiple myeloma	NK-92	ScFv-CD3ζ	Lentivirus	[89]	2014
CS-1	multiple myeloma	NK-92	scFv-CD28-CD3ζ	Lentivirus	[90]	2014
BCMA	multiple myeloma	NK-92	scFv-4-1BB-CD3ζ	Lentivirus	[91]	2019
BCMA	multiple myeloma	PB	ScFv-CD3ζ/DAP12	mRNA electroporation	[92]	2022
CD38	AML	PB	scFv-CD28-CD3ζ	mRNA electroporation	[93]	2022
CD38	AML	PB	scFv-CD28-CD3ζ	mRNA electroporation	[68]	2020
CD33	AML	PB	scFv-4-1BB-CD3ζ	Lentivirus	[94]	2022
CD5	T-cell malignant cells	NK-92	scFv-CD28-4-1BB-CD3ζ	Lentivirus	[95]	2017
CD5	T-cell malignant cells	NK-92	scFv-2B4-CD3ζ	Lentivirus	[96]	2019
CD7	T-cell Leukemia	NK-92	scFv-CD28-4-1BB-CD3ζ	mRNA electroporation	[97]	2019
CD123	AML	PB	scFv-CD28-4-1BB-CD3ζ	Retrovirus	[98]	2017
CD123	AML	NK-92	scFv-CD28-4-1BB-CD3ζ	Retrovirus	[99]	2021
NKG2DL	AML	PB	NKG2D-CD28-4-1BB-CD3ζ	mRNA electroporation	[100]	2021

**Table 3:** Targets of CAR-NK cell-based therapy for solid tumors

Target	Tumor	NK cell sources	CAR structures	CAR transfer	Reference	Year
HER2	Breast and Ovarian carcinoma	PB	scFv-CD28-CD3ζ	Retrovirus	[101]	2008
HER2	Breast cancer	NK-92	scFv-CD28-CD3ζ	mRNA electroporation	[102]	2015
HER2	Breast cancer and Renal cell carcinoma	NK-92	scFv‐CD3ζ/scFv-CD28-CD3ζ/scFv-4-1BB-CD3ζ	Lentivirus	[103]	2015
HER2	Glioblastoma	NK-92	scFv-CD28-CD3ζ	Lentivirus	[104]	2016
HER2	Gastric cancer	NK-92	scFv-4-1BB-CD3ζ	Lentivirus	[105]	2019
EGFR/EGFRvIII	Glioblastoma	NK-92/NKL cells/PB	scFv-CD2-CD3ζ	Lentivirus	[76]	2015
EGFR/EGFRvIII	Glioblastoma	NK cell line YTS	scFv-DAP12 + CXCR4	Lentivirus	[106]	2015
EGFR/EGFRvIII	Breast cancer/Brain metastases	NK-92/PB	scFv-CD28-CD3ζ	Lentivirus	[107]	2016
EGFR/EGFRvIII	Glioblastoma	PB	scFv-CD28-CD3ζ	Retrovirus	[108]	2021
EGFR/EGFRvIII	Breast cancer	PB	scFv-CD28-4-1BB-CD3ζ	Lentivirus	[109]	2020
GD2	Neuroblastoma	NK-92	scFv-CD3ζ	Retrovirus	[110]	2012
GD2	Ewing sarcomas	PB	scFv-4-1BB-2B4-CD3ζ	Retrovirus	[111]	2016
Mesothelin	Ovarian cancer	iPSC-NK cells/NK-92	scFv-NKG2D-2B4-CD3ζ scFv-NKG2D-2B4-DAP10-CD3ζ scFv-NKG2D-4-1BB-2B4-CD3ζ	Transposon transfection	[33]	2018
Mesothelin	Ovarian cancer	NK-92	scFv-CD28-4-1BB-CD3ζ	Lentivirus	[112]	2020
Mesothelin	gastric cancer	NK-92	scFv-NKG2D-2B4-CD3ζ	Lentivirus	[113]	2021
PSCA	Prostate cancer/Bladder carcinoma/Glioblastoma	NK cell line YTS	scFv-DAP12/scFv-CD3ζ	Lentivirus	[40]	2015
PSMA	Prostate cancer	NK-92	scFv-CD28-CD3ζ	Lentivirus	[114]	2020
PSMA	Prostate cancer	NK-92	scFv-NKG2D-2B4-CD3ζ	Lentivirus	[115]	2022
Glypican-3	Hepatocellular carcinoma	NK-92	scFv-CD28-CD3ζ	Lentivirus	[116]	2018
Glypican-3	Ovarian cancer	iPSC-NK	scFv-CD28-4-1BB-CD3ζ	Lentivirus	[117]	2020
Glypican-3	Hepatocellular carcinoma	NK-92	scFv-CD3ζ/scFv-CD28-CD3ζ/ scFv-DNAM1 or 2B4-CD3ζ	Lentivirus	[118]	2020
EpCAM	Breast carcinoma	NK-92	scFv-CD28-CD3ζ + IL-15	Lentivirus	[119]	2012
HLA-G	Solid tumor	PB	scFv-KIR2DS4-DAP12 + iCasp9	Lentivirus	[120]	2021
GPA7	Melanoma	NK-92	scFv-CD3ζ	mRNA electroporation	[121]	2017
c-MET	Hepatocellular carcinoma	PB	scFv-4-1BB-DAP12-EGFRt-V5	Lentivirus	[122]	2019
B7-H3	Non-small cell lung cancer	NK-92	scFv-4-1BB-CD3ζ	Lentivirus	[123]	2020
DLL3	Small cell lung cancer	NK-92	scFv-NKG2D-2B4-CD3ζ	Lentivirus	[124]	2022

## Advantages of CAR-NK immunotherapy

### Abundant sources of NK cells

Compared with CAR-T cells that can only be derived from autologous (patient-derived) peripheral blood, CAR-NK cells have more diverse sources to choose from. Different cell sources and corresponding procedures for manufacturing CAR-NK cell products were summarized in the Fig. 1C. Allogeneic NK cells provide a greater possibility of making “off-the-shelf” products [125], which can not only reduce the long period needed for CAR-T production, but also prevent patients with poor physical condition from being unable to receive CAR-engineered cellular immunotherapy. Because patients whose immunity is already severely damaged may not provide enough sufficient functional lymphocytes. Thus, CAR-NK immunotherapy may be more applicable than CAR-T cells in the future. CAR-NK cells can be obtained from several different sources of NK cells, including peripheral blood-derived NK (PB-NK) cells; cord blood-derived NK (CB-NK) cells; some clonal cell lines such as KHYG-1, NK-92, NKL and YT cells; NK cells derived from human induced pluripotent stem cells (iPSC-NK) or human embryonic stem cells [47, 69, 126, 127].

PB-NK and CB-NK cells are both primary NK cells, which need to be activated and expanded before the cytotoxicity of CAR-NK therapy can be effectively exerted [128]. Many clinical trials used PB-NK cells as they are derived from HLA-mismatched donors with no risk of GVHDs [129]. NK cells can be isolated from peripheral blood mononuclear cells by CD3 depletion or combining consecutive CD56-positive selection of NK cell isolation kits [130–132]. Then they can be expanded and activated in NK cell-specific expansion media with cytokines and other stimulated cells suitable for clinical use [133]. It has been proved that a large number of highly active NK cells can still be obtained from frozen CB compared with fresh CB [134, 135]. Therefore, sufficient CB can be obtained at one time from the CB bank to produce large-scale CAR-NK cells, without temporary screening of a single qualified adult donor and leukapheresis as the PB-NK. In addition, the CB bank provides another strength for selecting NK cells of donors with certain HLA types and specific NK cell receptor characteristics. Selecting a donor with HLA-KIR mismatch may enhance the alloreactivity of NK cells [136, 137]. Therefore, compared with PB-NK, CAR-NK cells derived from CB-NK are more likely to serve as “off-the-shelf” products for cellular immunotherapy. However, some researchers found that CB-NK cells have immature phenotypes, exhibiting normal levels of degranulation but lower cytotoxicity, with decreased expression of some molecules such as CD16 compared with PB-NK cells [138–140].

NK cell lines are also the main source of CAR-NK cells in preclinical and clinical studies as they are relatively easy to successfully transduce CAR genes [45]. Among these cell lines, NK-92 is the most commonly used and the only one approved by the FDA for clinical trials of CAR-NK-92 therapy [141]. Tang reported a first-in-man clinical trial of CD33-CAR NK-92 cells and showed that this therapy can be safely used in patients with relapsed and refractory AML [142]. There are a series of activated receptors but almost no inhibitory killer receptors expressed on NK-92 cells, and NK-92 cells are unable to mediate ADCC because of shortage of the CD16 receptor [143–145]. CAR-engineered NK-92 cells have strong cytotoxicity and can obtain a large number of cells with the same phenotypes in a short time [86, 146]. The disadvantage of CAR-NK92 cells is that they must be irradiated to avoid malignant proliferation before infused into patients [83]. The proliferation of irradiated CAR-NK cells is inhibited in vivo, which leads to a short persistence time [114]. Although it can avoid some side effects of CAR-T therapy such as “off-target effects,” it also reduces its anti-tumor effect, thereby requiring multiple transfusions.

NK cells can also be generated from hematopoietic progenitor cells (HPCs). Peripheral blood apheresis after stimulation, CB, bone marrow, and ESC are the approaches for obtaining CD34^+^HPC [147, 148]. After induced by the culture medium including a mixture of cytokines such as IL-3, stem cell factor, IL-15, FLT3L and IL-7, CD34^+^HPC will differentiate into NK cells for adoptive immunotherapy [149]. IPSC-NK is considered to be a potential cell source for “off-the-shelf” CAR-NK products because of its unlimited expansion ability and higher transduction efficiency than primary NK cells [33, 150, 151]. After proper ways of differentiation and expansion, only one CAR-iPSC cell can be used to obtain a large number of CAR-NK cells with uniform phenotypes. That's why it is easier to get a standardized “off-the-shelf” product. However, similar to CB-NK, iPSC-NK cells have lower cytotoxicity due to their immature phenotypes such as low CD16 and KIR expression, and high NKG2A expression compared with PB-NK cells [151–153]. Therefore, some problems need to be addressed before iPSCs can be used on a large scale in the future. The first clinical trial about CAR-engineered iPSC-NK product, FT596, was carried out in 2019. These CD19-targeted CAR iPSC-NK cells express a high-affinity, non-cleavable CD16 with an IL-15 receptor fusion protein. Therefore, their anti-tumor ability was enhanced by overcoming the low expression of CD16 and improving the expansion and persistence of CAR-NK cells in vivo. The ongoing phase I trial (NCT04245722) also provides a promising guidance for CAR-iPSC-NK to become “off-the-shelf” products for clinical cellular immunotherapy.

### Better safety and multiple available mechanisms of cytotoxicity

In contrast to CAR-T cell-based immunotherapy, CAR-NK cells have superior safety performance mainly reflected in the following aspects. First, allogeneic NK cells do not cause a lethal risk of GVHD that extremely associated with T cells, which has been verified in some animal or human clinical trials of CAR-NK cells [154, 155]. In addition, the cytokines secreted by NK cells are almost GM-CSF and IFN-γ with lower toxicity profiles [156]. They are different from most inflammatory cytokines secreted by T cells such as IL-1, IL-2, IL-6, TNF-a, IL-8, IL-15, MCP1, and IL-10, which are closely related to the side effects of CAR-T therapy [157, 158]. Therefore, CAR-NK cells injected into the patient nearly have no side effects such as CRS or ICANS, making them become a more attractive choice for anti-tumor cellular immunotherapy. In addition, the lifespan of NK cells in the blood cycle is relatively limited [159], further reducing the potential for toxicity to normal tissue, such as B cell aplasia caused by long-term persistence of CAR-T cells in vivo [160]. But it is possible that designed CAR structures will induce unanticipated toxicity due to excessive cytokine production [161]. Considering the potential toxicity caused by long-lived genetically modified CAR-NK cells, suicide genes have been used as safety switches and inducible caspase 9 (iCas9) has been shown to effectively eliminate CAR-NK cells both in vitro and in vivo [162, 163].

As mentioned earlier, NK cells release cytotoxicity depending on their germline-encoded activating and inhibitory receptors. And they do not rely on the existence of tumor-associated antigens on tumor cells. Therefore, even if tumor cells initiate immune escape mechanisms and lead to the down-regulation of the targeted antigens, CAR-NK cells can still reserve the intrinsic killing efficiency mediated by their receptors. Some studies have also attempted to add a fusion protein of CD16 into the CAR structure to enhance ADCC [164, 165]. A high-affinity, non-cleavable CD16 (hnCD16) expressed on NK cells with CAR structures provides the possibility of combining CAR-NK cells with antibodies targeting different antigens. An iPSC-derived CAR-NK cell product, iDuo NK cell, is engineered with three functional elements including a CD19-CAR, a high-affinity and non-cleavable CD16, and a membrane-bound IL-15/IL-15R fusion molecule (IL-15RF). The modified iDuo NK cells exhibited effective elimination of both CD19^+^ and CD20^+^ lymphoma cells through a combination of intrinsic cytotoxicity, anti-CD19 CAR mediated killing, and ADCC mediated by hnCD16 engagement with rituximab [166]. Thus, unlike CAR-T cells that only rely on CAR-targeted mechanism, CAR-NK cells keep immune-monitoring and killing tumor cells that express different levels of CAR-targeted antigens through multiple cytotoxic mechanisms.

### High feasibility of “off-the-shelf” products

Simplifying the manufacturing process and saving cost are also major difficulties that should be overcome for the clinical widespread of CAR-T therapy. Many studies made efforts to develop efficient and reliable “off-the-shelf” T cell products. However, the requirements of individual specificity for autologous CAR-T cells, demand for facilities for cold-chain transportation, and unavoidable GVHD risks make it difficult to achieve. Unlike CAR-T cells, CAR-NK cells are regarded as an ideal alternative platform for cancer immunotherapy by researchers because of their unique advantages and the potential for the production of “off-the-shelf” immunotherapeutic products. For example, NK-92 cells engineered to express different CARs targeting CD19, CD20, CD38, HER2, PSMA or GD2 were considered as allogeneic “off-the-shelf” immunotherapeutic products and held great promise for the development of effective anti-cancer treatments [114, 144, 167, 168]. Moreover, CAR-NK cells obtained from iPSC are a standardized homogeneous cell population that can be promoted in a clinically scalable manner [151, 169]. With abundant sources, no risk of GVHD response, and no need for patient-specificity, CAR-NK cells are more feasible to steadily acquire “off-the-shelf” products.

### CAR-NK cells against solid tumors

Although there are large similarities between CAR-NK and CAR-T cell therapies and they are both known to be effective against hematological malignancies, the inefficiency of CAR-T cells in solid tumors should not be extended to CAR-NK therapy. The natural properties of NK cells and multiple killing mechanisms offer a promising prospect for CAR-NK cells in solid cancer therapy. A growing number of studies have examined the activity of CAR-NK cells against solid tumors, such as glioblastoma, breast, ovarian and pancreatic cancer [170]. Intracranial injections of bispecific EGFR- and EGFRvIII- targeted CAR-NK-92 prolonged the survival of glioblastoma xenograft mouse [171]. Several studies have demonstrated the effectiveness of CAR-NK cell therapies targeting mesothelin, CD24 and glypican-3 in ovarian cancer models [112, 117, 172]. Additionally, the first clinical trial of CAR-NK therapy for solid tumor treatment used MUC-1 CAR-NK cells to target against multiple malignant solid tumors, e.g., glioblastoma, pancreatic, colorectal, breast and ovarian cancer (NCT02839954). Of the eight patients, seven achieved stable disease without serious adverse events. Although CAR-NK therapy has a bright potential for solid tumor, there are still many limitations to overcome. Furthermore, the application of CAR-NK therapy for solid tumors may require additional modifications of the NK cells beyond CAR transduction to improve intratumor trafficking, overcome the immunosuppressive TME and prevent tumor antigens escape.

### Current clinical trials of CAR-NK cell immunotherapy

As of June 2022, there have been 32 clinical trials (summarized in Table 4) of CAR-NK therapy registered on the ClinicalTrials.Gov website. Two of them have been completed, nine of them are in early phase I, and 21 of them are in phase I/II. Among these trials, there are some targets for hematological malignant tumors, such as CD19, CD7, BCMA, CD33, CD22. Eleven trials are about solid tumors (including ovarian cancer, prostate cancer, non-small cell lung cancer, pancreatic cancer, and glioblastoma), in which CAR-NK cells target some over-expressed antigens such as MUC1, PSMA, ROBO1, mesothelin, and HER2. According to the data disclosed, PB-NK (8/32) and NK-92 (9/32) are the most common cell sources, and lentiviral transduction is mostly used (not shown in the table). To date, the results of a few clinical trials have been published. Two are small-scale (n = 3) clinical trials, using NK-92 targeting CD33 (NCT02944162) and PB-NK cells targeting NKG2DL (NCT03415100) [63, 142]. Both the small-scale clinical trials demonstrated the advantages of CAR-NK in the treatment of tumors, such as not inducing GVHD or other immune toxicities, providing the potential of “off-the-shelf” products for cancer immunotherapy.

**Table 4:** Current clinical trials of CAR-NK registered on ClinicalTrials.Gov

NCT number	Cancer type	NK source	Target	CAR structure	Country	Phase	Initial year	State
NCT04639739	non-Hodgkin lymphoma	Unknown	CD19	Unknown	China	Early phase 1	2020/11	Not recruited
NCT03692637	Epithelial ovarian cancer	PB-NK	Mesothelin	Unknown	China	Early phase 1	2018/10	Not recruited
NCT03692663	Castration-resistant prostate cancer	Unknown	PSMA	Unknown	China	Early phase 1	2018/10	Not recruited
NCT03692767	Refractory B-cell Lymphoma	Unknown	CD22	Unknown	China	Early phase 1	2018/10	Not recruited
NCT03690310	Refractory B-cell lymphoma	Unknown	CD19	Unknown	China	Early phase 1	2018/10	Not recruited
NCT03824964	Refractory B-cell lymphoma	Unknown	CD19/CD22	Unknown	China	Early phase 1	2019/1	Unknown
NCT03415100	NKG2D-ligand expressing solid cancer(metastatic solid tumors)	PB-NK	NKG2D-L	ScFv-CD8a_TM_-CD3ζ ScFv-CD8a_TM_-DAP12	China	Phase 1	2018/1	Recruited
NCT00995137	B-ALL	PB-NK	CD19	ScFv-CD8a_TM_-CD137-CD3ζ	USA	Phase 1	2009/10	Completed
NCT03383978	Glioblastoma	NK-92	Her2	ScFv-CD28-CD3ζ	Germany	Phase 1	2017/12	Recruited
NCT04245722	B-cell lymphoma or chronic lymphocytic leukemia	iPSC (FT596)	CD19	scFv-NKG2D-2B4-CD3ζ-IL-15/R-hnCD16	USA	Phase 1	2020/1	Recruited
NCT03940833	Multiple myeloma	NK-92	BCMA	Unknown	China	Phase1/Phase 2	2019/5	Recruited
NCT03940820	ROBO1 expression in solid tumor	NK-92	ROBO1	Unknown	China	Phase1/Phase 2	2019/5	Recruited
NCT02944162	CD33 + acute myeloid leukemias	NK-92	CD33	ScFv-CD28-CD137-CD3ζ	China	Phase1/Phase 2	2016/10	Completed
NCT02742727	Leukemia and lymphoma	NK-92	CD7	ScFv-CD28-4-1BB-CD3ζ	China	Phase1/Phase 2	2016/4	Recruited
NCT03579927	B-cell lymphoma	Cord blood	CD19	CD19-CD28-zeta-2A-iCasp9-IL15	USA	Phase1/Phase 2	2018/7	Withdrawn
NCT02839954	MUC1 positive relapsed or refractory solid tumor	NK-92	MUC1	ScFv-CD28-4-1BB-CD3ζ	China	Phase1/Phase 2	2016/7	Unknown
NCT02892695	Lymphoma, leukemias	NK-92	CD19	ScFv-CD28-4-1BB-CD3ζ	China	Phase1/Phase 2	2016/9	Unknown
NCT03056339	B-cell lymphoma	Cord blood	CD19	iCasp9-ScFv-CD28-CD3ζ-IL-15	USA	Phase1/Phase 2	2017/2	Recruited
NCT03941457	Pancreatic cancer	NK-92	ROBO1	Unknown	China	Phase1/Phase 2	2019/5	Recruited
NCT05410717	Claudin6 expressed solid tumor	PB-NK	Claudin6	Unknown	China	Phase1/Phase 2	2022/6	Recruited
NCT05213195	Colorectal Cancer	Unknown	NKG2D-L	Unknown	China	Phase 1	2021/12	Recruited
NCT05008536	Multiple Myeloma	Cord blood	BCMA	Unknown	China	Early phase 1	2021/10	Recruited
NCT05247957	NKG2DL expressed AML	Cord blood	NKG2D-L	Unknown	China	Phase 1	2021/10	Recruited
NCT05215015	AML	Unknown	CD33/CLL1	Unknown	China	Early phase 1	2021/11	Recruited
NCT05008575	AML	Unknown	CD33	Unknown	China	Phase 1	2021/12	Recruited
NCT05194709	Advanced Solid Tumors	Unknown	5T4	Unknown	China	Early phase 1	2021/12	Recruited
NCT04887012	B-cell NHL	PB-NK	CD19	Unknown	China	Phase 1	2021/05	Recruited
NCT05020678	B-cell malignancies	PB-NK	CD19	CD19ScFv-CD8a_TM_-OX40-CD3ζ-T2A-IL15	USA	Phase 1	2021/08	Recruited
NCT04847466	Gastric or Head and Neck Cancer	NK-92	PD-L1	Unknown	USA	Phase 2	2021/12	Recruited
NCT04623944	AML or MDS	PB-NK	NKG2D-L	ScFv-CD8a_TM_-OX40-CD3ζ-T2A-IL15	USA	Phase 1	2020/09	Recruited
NCT05410041	B-cell malignancies	PB-NK	CD19	Unknown	China	Phase 1	2022/05	Recruited
NCT05182073	Multiple Myeloma	iPSC (FT576)	BCMA	scFv-NKG2D-2B4-CD3ζ-IL-15/R-hnCD16	USA	Phase 1	2021/11	Recruited

Another one is a promising large-scale (NCT03056339) phase I/II clinical trial, which was published in February 2020. In this large-scale trial, 11 patients with R/R CD19-positive cancers (non-Hodgkin’s lymphoma or chronic lymphocytic leukemia) received an allogeneic CB-derived CAR-NK cell product after lymphodepleting chemotherapy [173]. Anti-CD19 CAR-NK cells were transduced to express IL-15 and an iCaspase-9-based safety switch. Eight of the 11 patients (73%) received treatment had no obvious side effects, such as CRS, ICANS and GVHD, seven patients had complete remission. The corresponding preclinical study reported that CB-derived NK cells were transduced with the genes for CAR-CD19 and IL-15. These cells showed effective cytotoxicity and apparent prolongation of survival with IL-15 production by the transduced CB-NK cells in a xenograft Raji lymphoma murine model [82]. In this clinical trial, expansion was observed as early as 3 days after injection. And CAR-NK cells persisted for at least 12 months, which was also associated with the incorporation of IL-15 in the CAR vector. In summary, the small progress of clinical trials indicates a bright future for the clinical application of CAR-NK products.

### Obstacles to the popularization of CAR-NK therapy and corresponding coping strategies

Regardless of the great prospect of CAR-NK cells in tumor immunotherapy, there are still some thorny problems hindering their further approval and promotion. Researchers are constantly exploring how to improve them for their successful popularization in the clinic.

### Expansion and purification of NK cells

A large-scale highly cytotoxic NK cells are needed for clinical adoptive therapy. The proportion of NK cells in peripheral blood lymphocytes does not exceed 10–15%, so in vitro culture for expansion is needed. Some cytokines can improve the expansion of NK cells, but it is very difficult to induce a constant proliferation of human NK cells under a kind of cytokine such as IL-2 [174]. Some researchers have shown that IL-2 in combination with other cytokines (anti-CD3 antibody or IL-15) can exert better stimulatory effects on NK cells [175–177]. Chronic myelogenous leukemia-derived cell line K562 can stimulate NK cells [178], but the unmodified K562 cell is of limited use. It needs to be modified before it can be used to amplify NK cells. Co-culturing PB-NK cells with K562 feeder cells genetically modified to express IL-15 and the adhesion molecule 4-1BBL is a good method [78]. A manually performed experiment with GMP-compliant media containing IL-2, IL-21 and irradiated autologous feeder cells made an effective cell expansion, which induced an 85-fold NK cell expansion [98]. Recently, Yan Yang et al. applied a new feeder cell system (human B-lymphoblastoid cell-line 721.221) with human B-lymphoblastoid cell-line 721.221 to stimulate NK cells. The results showed that new feeder cells were superior to K562-mIL-21 cells with better proliferation and less apoptosis during expansion [179]. NK cells from human ESC and iPSC have been certified to have functions similar to conventional primary cells. But they are more homogenous and feasible to be genetically modified at a clonal level, and easy to be expanded in a clinical scale [139, 153, 180]. Another trouble is allogenic NK cells may be contaminated with T cells. In a first-in-human trial of adoptive transfer of donor-derived IL-15/4-1BBL-activated NK cells (aNK), researchers found that aNK-DLI contributed to acute GVHD, likely by augmenting the underlying T-cell alloreactivity [181]. Pre-lymphocyte deletion may contribute to purifying NK cells [182]. Moreover, regulatory T cells and myeloid-derived suppressor cells (MDSCs) may influence NK cells therapies [45]. It is necessary that finding a suitable method to expand and purify NK cells to serve the clinic effectively.

### *Persistence of CAR-NK cells *in vivo

NK cells do not go through massive expansions and have a limited lifespan in vivo, which is why they do not cause severe side effects unlike CAR-T cells but result in low persistence. Cytokines such as IL-2 and IL-15 are key molecules involved in NK cells functions, including differentiation, proliferation, activation, and survival [183, 184]. IL-2 diphtheria toxin fusion protein (IL-2DT) may improve the expansion of NK cells in AML patients [185]. However, a high dose of IL-2 can cause serious adverse effects [186]. IL-15 has been demonstrated to stimulate NK cells proliferation and up-regulate activating receptor NKp30 expression on NK cells in vitro [82, 187, 188]. ALT-803, a kind of superagonist, was shown to be well tolerated by patients and could promote CD8^+^ T and NK cell expansion in vivo [188]. A TriKE (IL-15 trispecific killer engager that targets CD33) was constructed to induce expansion and persistence in vivo with a modified IL-15 cross-link [189, 190]. Currently, TriKE is being evaluated in phase I/II clinical trials in patients with CD33-expressing high-risk myelodysplastic syndromes, such as R/R AML (NCT03214666).

### Suppression of TME

After being attacked, tumor cells may directly inhibit the functions of CAR-NK cells or contribute to the generation of a local suppressive TME. This is the reason why CAR-NK cells, with high cytotoxicity against cancer cells, gradually lose their anti-tumor ability in vivo.

Tumor cells can produce some soluble immune factors such as TGF-β, prostaglandin E2 (PGE2), indoleamine 2,3-dioxygenase (IDO), IL-10, and PD-1, which suppress the function of NK cells. Produced by neutrophils, macrophages, and Tregs in the TME, TGF-β may affect the metabolism of NK cells and down-regulate NKG2D and NKp30 expression [191, 192]. Tregs and immunosuppressive MDSCs are actively recruited to the local TME to inhibit the cytotoxicity of CAR-NK cells and promote tumor growth [193]. PGE2 has a negative impact on the differentiation and cytotoxicity of NK cells, and inhibits expression of NKp44 and NKp30 on NK cells. [194, 195].

TME is frequently characterized by hypoxia and it is a common metabolic disturbance. It can greatly influence NK cells by affecting their metabolism and infiltration, cytokines production, and expression of several activating receptors [196–198]. Activating receptors such as NKp30, NKp46, NKp44, and NKG2D on NK cells would be down-regulated for tumor growth and metastasis [197]. CD73 induces the expression of arginase, an immunosuppressive metabolite. Arginase is produced under hypoxia conditions and inhibits NK cell functions. The inhibition of CD73 increases the homing of NKG2D-CAR NK cells that target tumor cells expressing NKG2D ligands and improves anti-tumor responses in animal models of lung cancer [199]. In terms of migration, hypoxia may also influence the surface expression of CCR7 and CXCR4 on CD56 bright NK cells, increasing their migration response to CCL19/21 and CXCL12 [198].

Tumor cells can also express ligands for the so-called checkpoint proteins that help tumor cells evade immune surveillance. PD-1 has been verified to be expressed in PB-NK cells from patients with multiple myeloma, and in blood and tumor-associated NK cells from patients with renal and ovarian cancers [200–202]. CAR constructions combined with blockers of checkpoint proteins such as anti-PD-1, CTLA-4, LAG3, and TIGIT have more therapeutic benefits compared with using traditional CAR structures [203]. NK-92 cells engineered to express high-affinity CD16, IL-2, and PD-L1-specific CAR structure released high levels of perforin and granzyme and lysed human cancer cell lines including breast, lung, and gastric cancers [204].

### Storage, shipping, and recovery of “off-the-shelf” CAR-NK cells

The storage, shipping, and recovery of “off-the-shelf” CAR-NK cells are necessary to facilitate large-scale clinical promotion, but some problems come across during these processes. Compared with T cells, NK cells are more sensitive to the process of freezing and thawing. The survival rate and cytotoxicity of NK cells are significantly down-regulated after thawing [205]. Some researchers found that the influence of frozen NK cells can be improved by culturing with IL-2 [206]. Moreover, cytokine-activated NK cells are very sensitive to lower temperature [207]. Their cytotoxicity should be maintained under body temperature during the process of shipping. Appropriate cell density is also crucial in the process of shipping. High cell concentrations may lead to the loss of cells activity possibly due to the rapid consumption of medium and changes in glucose and pH value. Moreover, the quality of cells is difficult to control in the shipping process. The sensitivity of NK cells to cryopreservation makes them difficult to store and transport, which is a limitation of CAR-NK cell therapy. Strategies for optimal cryopreservation must be explored to make CAR-NK cells therapy available for “off-the-shelf” products.

### Combination strategies of CAR-NK cells with other therapies

NK cells express CD16 and exert tumor killing through ADCC. Another strategy for combination therapy is to combine CAR-NK cells with antibodies targeting different tumor antigens. The ADCC-inducing antibodies of anti-CD20, anti-HER2, anti-EGFR and anti-GD2 have promising results on refractory NHL, breast cancer, colorectal cancer and neuroblastoma [208]. FT596 not only targeted CD19^+^ lymphoma, but also exhibited enhanced killing effect on the CD20^+^ lymphoma cells when combined with the anti-CD20 antibody rituximab (NCT04245722). IL-2 and IL-15 have been identified as key cytokines that upregulate the activity of NK cells. The antitumor effect of PD-L1 CAR-NK cells, in combination with anti-PD-1 and N-803, an IL-15 superagonist, resulted in superior control of tumor growth in C57BL/6 mice [204]. Therefore, cytokines-based treatment may improve the persistence and cytotoxicity of CAR-NK cells. In addition, chemotherapy not only reduces rejection of infused allogeneic NK cells by the recipient, but may also reprogram the TME to facilitate NK cell infiltration and survival in vivo [209]. The sequential treatment with chemotherapeutic agent followed by CAR-NK cells led to the strongest clinical efficacy of ovarian cancer [210]. Chemotherapy remains an important adjuvant therapeutic approach for future CAR-NK cell therapy.

Blockade of PD-1/PDL1 axis in combination with CAR-T therapy has been proved to be effectively in improving the anti-tumor effect [211, 212]. PD-1 disruption by CRISPR/Cas9 augments anti-CD19 CAR-T cell mediated tumor killing [213]. Similar to T cells, NK cells express some immune checkpoints molecules that inhibit the anti-tumor activity of activated NK cells. Immune checkpoint blockade (ICB) therapies by corresponding antibodies (e.g., anti-PD1/PDL1 and anti-CTLA4) that block inhibitory signals of NK cells activation can also enhance NK cell activity. Combination therapy of anti-PSMA CAR-NK cells and anti-PD-L1 monoclonal antibody enhances the anti-tumor efficacy against prostate cancer [115]. Studies have demonstrated that targeting ICB molecules like PD-1/PDL1 or B7-H3 can enhance the anti-tumor activity of CAR-NK cells [214, 215]. The silencing of immune checkpoint NKG2A enhances NK cell mediated cytotoxicity against multiple myeloma [216]. Therefore, CAR-NK cells in combination with ICB therapy or with knockout of ICB molecules by gene-editing will be a promising approach for improving the efficiency of CAR-NK therapy, especially for solid tumors.

## Conclusion and outlook

Currently, CAR-NK cell-based therapy has become a popular research field because of its unique advantages and feasibility of “off-the-shelf” products compared with CAR-T therapy, so it might become an alternative cellular immunotherapy for cancers. Combining CAR engineering with CD16 expression on NK cells also further enhances their anti-tumor ability by utterly utilizing their cytotoxic capacity and ADCC. However, some problems need to be studied and addressed continuously, including how to optimize the gene editing technology of CAR structure specific for NK cells, how to expand and activate NK cells briefly and effectively, and how to improve persistence and repair TME to advance the therapeutic effect of solid tumors. With the approval of more clinical trials and the guidance of more clinical data in the next few years, CAR-NK cells will be gradually optimized to provide efficient and safe “off-the-shelf” products for cancer immunotherapy.

## Data Availability

Not applicable.

## Abbreviations

CAR: Chimeric antigen receptor
NK: Natural killer
R/R: Relapsed/refractory
GVHD: Graft-versus-host disease
CRS: Cytokine release syndrome
ICANS: Immune effector cell-associated neurotoxicity syndrome
scFv: Single-chain variable fragment
ITAMs: Immunoreceptor tyrosine-based activation motifs
BaEV-gp: Baboon envelop glycoprotein
VSV-G: Vesicular stomatitis virus glycoprotein G
KIRs: Killer immunoglobulin-like receptors
HLA: Human leukocyte antigen
hnCD16: High-affinity, non-cleavable CD16
ADCC: Antibody-dependent cell-mediated cytotoxicity
PB: Peripheral blood
CB: Cord blood
iPSCs: Induced pluripotent stem cells
AML: Acute myeloid leukemia
HPCs: Hematopoietic progenitor cells
MDSCs: Myeloid-derived suppressor cells
TME: Tumor microenvironment
PGE2: Prostaglandin E2
